# Methodology for awakening the potential secondary metabolic capacity in actinomycetes

**DOI:** 10.3762/bjoc.20.69

**Published:** 2024-04-10

**Authors:** Shun Saito, Midori A Arai

**Affiliations:** 1 Department of Biosciences and Informatics, Keio University, 3-14-1 Hiyoshi, Kohoku-ku, Yokohama 223-8522, Japan

**Keywords:** actinomycete, co-culture, heat shock metabolites (HSMs), secondary metabolites, silent genes

## Abstract

Secondary metabolites produced by actinomycete strains undoubtedly have great potential for use in applied research areas such as drug discovery. However, it is becoming difficult to obtain novel compounds because of repeated isolation around the world. Therefore, a new strategy for discovering novel secondary metabolites is needed. Many researchers believe that actinomycetes have as yet unanalyzed secondary metabolic activities, and the associated undiscovered secondary metabolite biosynthesis genes are called “silent” genes. This review outlines several approaches to further activate the metabolic potential of actinomycetes.

## Introduction

Research clearly indicates that compounds derived from natural products have contributed greatly to enhancing the welfare of human society. For example, the structures of compounds produced by microorganisms are very diverse, facilitating their application in the development of therapeutic drugs [1], agrochemicals [2], veterinary medicines [3], medium supplements for selective microbial/cell culture [4–6], food preservatives/colorings [7–8], and biochemical reagents for pharmacological/chemical biology studies [9]. This review has focused particularly on compounds produced by actinomycetes. Secondary metabolites produced by actinomycetes, representing a class of compounds with a diverse chemical space, have promising potential as sources of bioactive substances [10]. Due to his discovery of streptomycin from *Streptomyces griseus*, Waksman was awarded the Nobel Prize in Physiology and Medicine in 1952 [11]. Ōmura, who discovered avermectin from *Streptomyces avermitilis*, received the same award in 2015 [12]. While there are many such brilliant achievements, researchers are finding it increasingly difficult to isolate novel compounds from secondary metabolites produced by actinomycetes [13–14]. This is the result of more than 50 years of effort to scrutinize substances produced by actinomycetes. However, many researchers have questioned whether the potential secondary metabolic capacity of actinomycetes has been fully analyzed and have continued to search for compounds from actinomycetes based on original ideas. For example, in searching for compounds based on biological activity, a proper screening system is critical [15]. Screening systems are broadly classified as either forward screening (searching for compounds that induce phenotypic changes) or reverse screening (searching for compounds that act directly on specific molecules). Imoto and colleagues constructed a reverse screening system that targets a malignant factor of prostate cancer and thereby identified several novel compounds from actinomycete strains [16–19].

**Figure 1:**
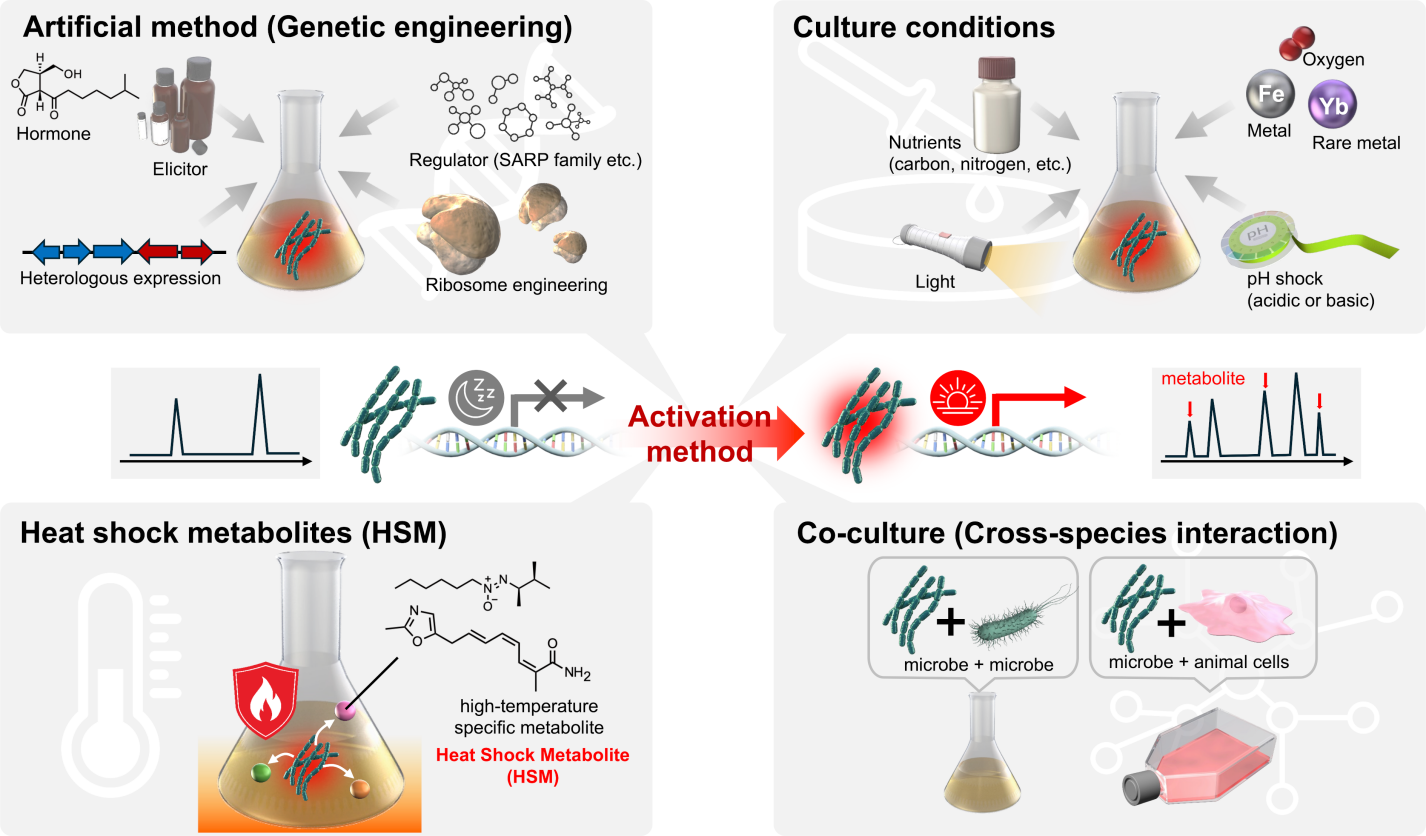
Schematic diagram of methods to activate silent genes in actinomycetes as presented in this review. This figure was created by Science Graphics Co., Ltd. This content is not subject to CC BY 4.0.

Many researchers have focused on identifying secondary metabolites from actinomycetes of the genus *Streptomyces* due to the prominence of species of this genus in surface soils and the difficulty of isolating members of other genera from natural environments. For this reason, actinomycetes other than those of the genus *Streptomyces* are referred to as “rare actinomycetes”, and the secondary metabolites produced by these species have thus attracted increasing attention. Indeed, an increasing proportion of newly identified antibiotics are reportedly produced by rare actinomycetes [20]. Igarashi and colleagues have also focused on various rare actinomycetes, analyzing their secondary metabolic activity and demonstrating the importance of studying new genera and species [21–25]. On the other hand, it is now thought that secondary metabolic activities remain to be analyzed in actinomycetes, including *Streptomyces* strains, which were considered to have been completely characterized. This may be because the number of discovered compounds is small compared to the number of secondary metabolite biosynthesis genes harbored by actinomycetes [26–28]. For example, in *Streptomyces avermitilis*, 38 secondary metabolite biosynthetic gene clusters have been identified by genome sequencing. Interestingly, only 16 secondary metabolites of *S. avermitilis* have been identified to date, even though the species is widely used as a standard actinomycete. This means that only 40% of the secondary metabolites from the 38 gene clusters have been identified. These as yet undiscovered secondary metabolite biosynthesis genes are called “silent genes”, because they are either not expressed or their expression levels are low under normal culture conditions. A number of studies have reported methods to activate these genes, and many new compounds have been discovered. In this review, we outline the silent gene activation methods, including the authors’ efforts (Figure 1).

## Review

### Artificial methods

**Figure 2:**
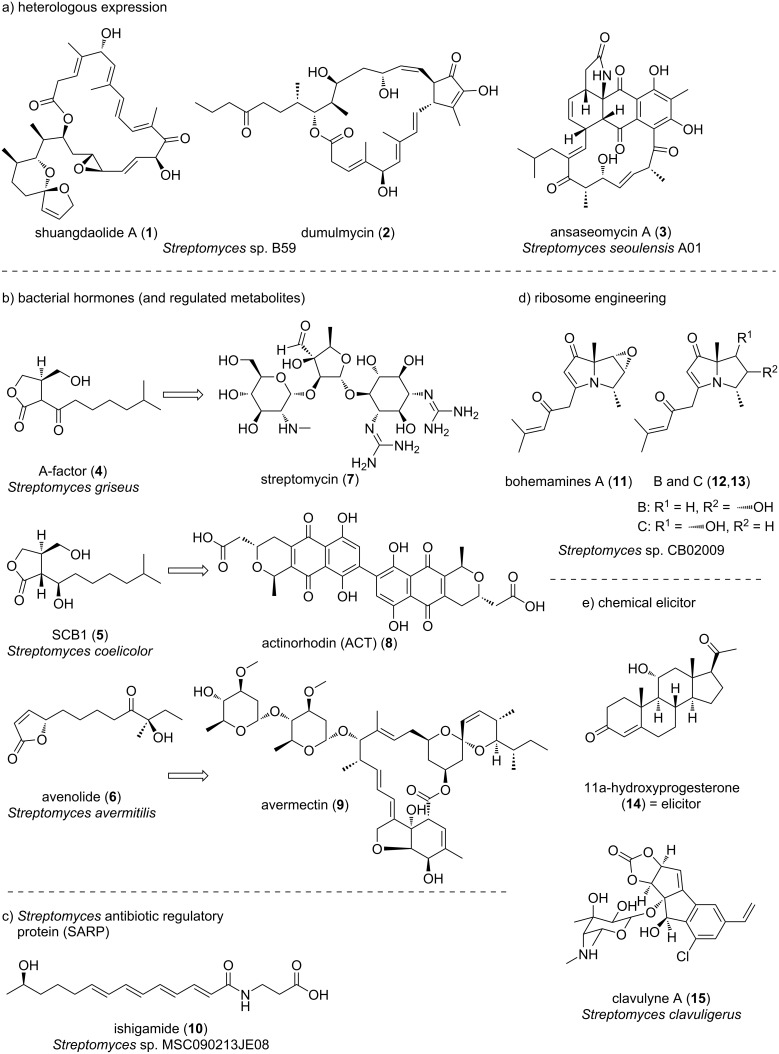
Structures of secondary metabolites obtained from actinomycetes using artificial methods.

Several target-oriented methods focusing on specific biosynthetic genes and regulatory factors and artificial methods have been reported to activate secondary metabolism in actinomycetes. The technique known as “heterologous expression”, in which a biosynthetic gene cluster of a secondary metabolite is introduced into another organism, is a useful method for producing silent secondary metabolites (Figure 2a). For example, the cryptic trans-acyltransferase polyketide synthase biosynthetic gene cluster *sdl* (80 kb) from *Streptomyces* sp. B59 was cloned and transferred into a heterologous host, *Streptomyces albus* J1074, resulting in the production of a class of polycyclic macrolide shuangdaolides A (**1**), B, and D and dumulmycin (**2**) [29]. Furthermore, genome mining of the marine actinomycete *Streptomyces seoulensis* A01 enabled the identification of a giant type I polyketide synthase gene cluster (*asm*) [30]. Heterologous expression of the cryptic *asm* cluster using a bacterial artificial chromosome vector in a heterologous host led to the production of ansaseomycins A (**3**) and B.

A number of factors associated with the biosynthetic gene clusters of secondary metabolites regulate their expression; thus, controlling these factors can also be used to activate secondary metabolism (Figure 2b). For example, the production of some secondary metabolites in actinomycetes is regulated by various bacterial hormones, such as A-factor (**4**) [31] and SCB1 (**5**) [32]. Normally, the tetR regulator negatively regulates the expression of biosynthetic genes in actinomycetes, but bacterial hormones release this repression [33–34]. A-factor, SCB1, and avenolide (**6**) induce the production of streptomycin (**7**), actinorhodin (**8**), and avermectin (**9**), respectively [35–37]. The production of these metabolites is regulated by the respective tetR regulators ArpA, ScbR, and AvaR1, respectively [38–40]. Therefore, increasing the levels of bacterial hormones present or disruption of the tetR regulator have been reported in several studies as methods to improve target-substance production capacity. Interestingly, Wilbanks et al. reported the structure–activity relationships of bacterial hormones in secondary metabolite biosynthesis by diversifiable synthesis [41]. The ability to synthesize stereochemically pure bacterial hormones would greatly expedite studies of these repressors and ultimately lead to the discovery of regulatory systems for many secondary metabolites.

Overexpression of transcription factors present in biosynthetic gene clusters can also be employed to increase the production of secondary metabolites. For example, overexpression of *Streptomyces* antibiotic regulatory protein (SARP) family transcriptional activators led to the discovery of many new secondary metabolites [42]. Du et al. searched for SARP family transcriptional activators from the draft genome of *Streptomyces* sp. MSC090213JE08, and constructed recombinant strains with forced expression of each of the seven SARP genes. When these were cultured in four different media and examined for metabolic profiles, a total of nine unknown metabolites were found in the four SARP-expressing strains under specific culture conditions. Du et al. identified a new compound (designated ishigamide (**10**)) as 3-([2*E*,4*E*,6*E*,8*E*]-13-hydroxytetradeca-2,4,6,8-tetraenamido)propanoic acid and elucidated the biosynthetic machinery (Figure 2c) [43–45]. Seo et al. synthesized ishigamide and determined its absolute configuration [46].

Finally, several studies have reported the modulation of secondary metabolic activation in actinomycetes using small molecules. For example, Ochi et al. proposed the “ribosome engineering” technique, in which actinomycetes are cultured with antibiotics to activate secondary metabolism by inducing resistance, and this approach has contributed to the isolation of many new compounds [47]. The ribosome engineering method is based on the principle that a mutation in the ribosomal S12 protein, which is associated with the acquisition of antibiotic resistance, activates secondary metabolism. The ribosomal S12 protein mutation results in increased expression of translation factors, which leads to enhanced protein synthesis in secondary metabolism [47]. Liu et al. reported activation of the production of bohemamines **11**–**13**, bacterial alkaloids containing a pyrrolizidine core with two unusual methyl groups, using a ribosome engineering approach in *Streptomyces* sp. CB02009 (Figure 2d) [48].

Seyedsayamdost et al. developed the high-throughput elicitor screening (HiTES) approach, a forward chemical genetics method that identifies small-molecule inducers of silent natural products (Figure 2e) [49]. Han et al. applied a fluorescence-based DNA cleavage assay coupled with HiTES to *Streptomyces clavuligerus* and identified the steroid 11α-hydroxyprogesterone (**14**) as an effective elicitor and characterized 10 cryptic enediyne-derived natural products, designated clavulynes A (**15**) and B–J with unusual carbonate and terminal olefin functionalities [50]. Thus, artificial methods of genetic engineering and chemistry also play an important role in the identification of novel secondary metabolites.

### Culture conditions

#### Medium composition, pH, oxygen supply, light

**Figure 3:**
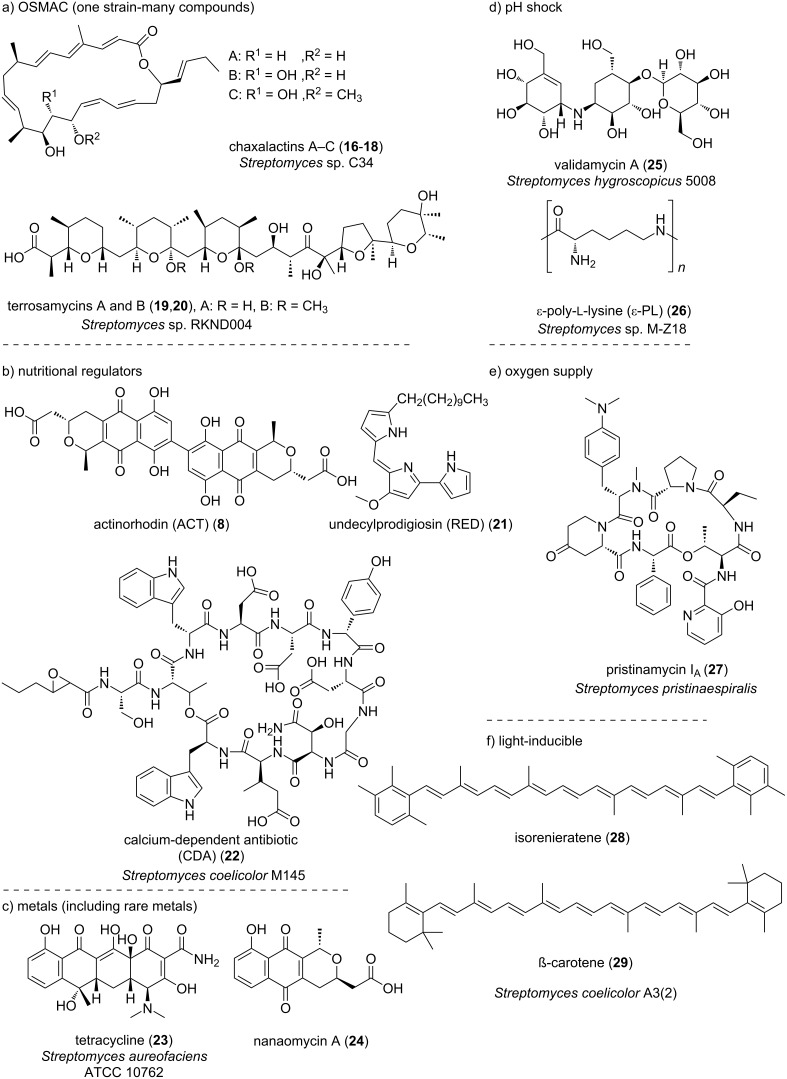
Structures of secondary metabolites obtained from actinomycetes by adjusting culture conditions.

Simply modifying the culture conditions is an important means of activating silent genes in actinomycetes. Because the cultivation conditions play a key role in metabolite production by actinomycetes, changing these parameters can significantly alter secondary metabolite production patterns. For example, OSMAC (one strain-multi compound), developed by Zeeck and co-workers in the early 2000s, is a method in which the target bacteria are cultured under various conditions (medium composition, temperature, pH, oxygen supply, light quality and quantity, addition of precursors and enzyme inhibitors, etc.) and all metabolites obtained from them are analyzed (Figure 3a) [51]. Zeeck's group has successfully isolated metabolites consisting of diverse structural classes from only a few microbial species by their OSMAC approach [52–54]. By applying this method, Rateb et al. and Sproule et al. discovered 22-membered macrolactone polyketides designated chaxalactins A–C (**16**–**18**), and polycyclic polyether natural products designated terrosamycins A and B (**19**, **20**), respectively [55–56]. Thus, the OSMAC strategy has been successfully applied over the last 10 years to generate new secondary metabolites from a single microbial strain.

Sensor proteins for medium components are also present in actinomycetes, and a regulatory system for secondary metabolism has been reported [57]. For example, Świątek et al. proposed that Rok7B7, a transcriptional regulator, acts as the central control protein for carbon control in *Streptomyces* [58–59]. Rok7B7 also controls the production of antibiotics such as actinorhodin (ACT, **8**), undecylprodigiosin (RED, **21**), and calcium-dependent antibiotic (CDA, **22**) in *Streptomyces coelicolor* M145 (Figure 3b). Chen et al. and Shu et al. reported that under stress associated with high concentrations of glutamate, AfsQ1/Q2 is important not only for maintaining the metabolic homeostasis of nutrient utilization but also for biosynthesis of antibiotics such as ACT, RED, CDA and the yellow pigment coelimycin P2 in *Streptomyces coelicolor* strains M145 [60–61].

Depletion of a metal component essential for growth activates the production of secondary metabolites with chelating activity, which enables the actinomycetes to acquire that metal (Figure 3c). Béchet and Blondeau reported that *Streptomyces aureofaciens* ATCC 10762 produces substantial amounts of tetracycline (**23**) only when the defined medium is deprived of iron [62]. Although not a compound obtained using approaches for activating silent genes, Wang et al. discovered SF2768, a metabolite produced by *Streptomyces thioluteus* that chelates copper as a chalkophore [63]. However, it has been suggested that activation of secondary metabolism in actinomycetes may depend on various metals. Indeed, it has been reported that adding to the culture medium rare metals not normally used as medium components can activate secondary metabolite biosynthetic genes. For example, Kamijo et al. found that *Streptomyces* sp. YB-1 produced reddish-purple pigment(s) only in the presence of a rare earth metal, ytterbium (Yb), but not in the presence of other metals. Structural analyses revealed that this pigment is a type of naphthoquinone similar to nanaomycin (**24**) [64]. Furthermore, Tanaka et al. showed that low concentrations of the rare earth elements scandium (Sc) and/or lanthanum (La) markedly activate (2.5 to 12-fold increase) the expression of nine secondary metabolite biosynthetic gene clusters in *Streptomyces coelicolor* A3(2) [65].

Furthermore, it has been reported that changing the pH of the culture medium can also result in the production of new substances (Figure 3d). Jiang et al. achieved a higher yield of validamycin A (**25**) from *Streptomyces hygroscopicus* 5008 together with more rapid protein synthesis and sugar consumption by subjecting cultures to one or more NaOH shocks [66]. Ren et al. obtained enhanced ε-poly-ʟ-lysine (ε-PL, **26**) production by acidic pH shock of *Streptomyces* sp. M-Z18, achieving 52.5% higher production than controls without pH shock [67]. Conversely, the present authors were able to increase the production of antarlides, which have a 22-membered ring macrocyclic structure, by adding disodium hydrogen phosphate to the culture medium of *Streptomyces* sp. BB47 to prevent the pH of the culture medium from becoming too acidic [17]. In addition, Mehmood et al. reported that the oxygen supply controls the production of pristinamycins **27** in *Streptomyces pristinaespiralis* (Figure 3e) [68]. Takano et al. discovered the light-induced production of carotenoid pigments, including isorenieratene (**28**) and β-carotene (**29**), in *Streptomyces coelicolor* A3(2) (Figure 3f) [69]. Two transcriptional regulators, LitR and LitS, have been proposed as playing central roles in this light-induction mechanism in actinomycetes.

Thus, each element in the culture environment is important for the activation of silent genes. The authors’ group has been working on methods to activate these genes by increasing the culture temperature above the normal level.

#### Temperature – heat-shock metabolites (HSMs) – metabolites for which production is activated by high-temperature culture

**Figure 4:**
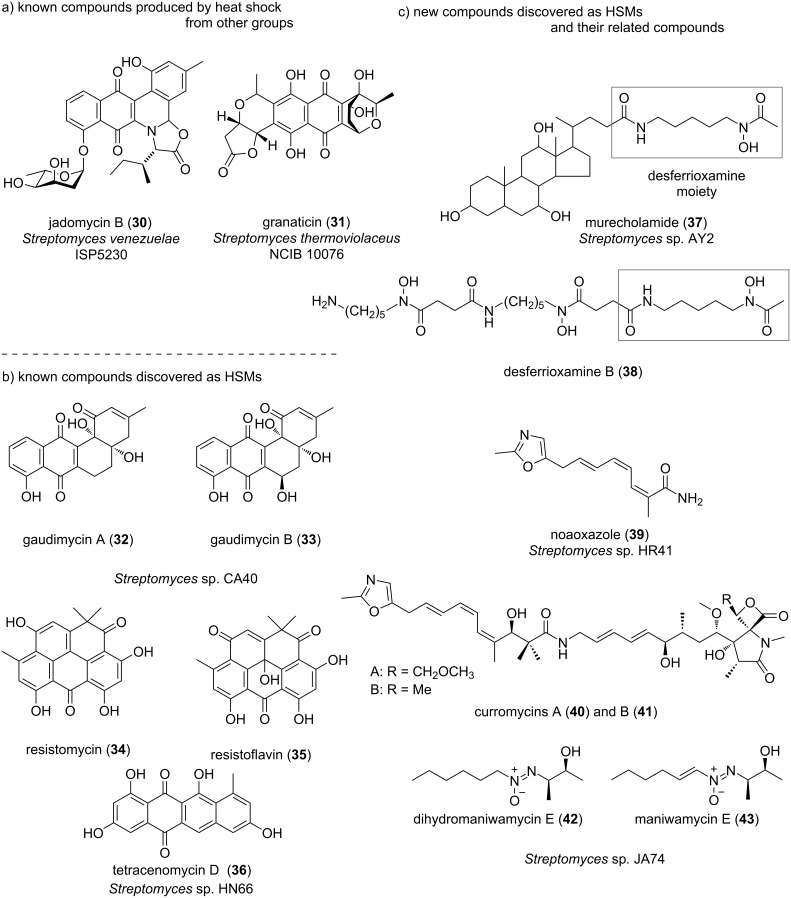
Structures of secondary metabolites obtained by high-temperature culture of actinomycetes.

Several examples of high-temperature culture of actinomycetes have been reported prior to the authors’ examples as studies attempting to increase the production of the targeted metabolites (Figure 4a). For example, Doull et al. achieved an increase in the production of jadomycin B (**30**) produced by *Streptomyces venezuelae* ISP5230 by heat shock at 42 °C for 1 h [70]. Similarly, James et al. increased the production of granaticin (**31**) by *Streptomyces thermoviolaceus* NCIB 10076 by changing the culture temperature [71]. They showed that the yield was best at 45 °C, whereas the rate of synthesis was highest at 37 °C. However, there have been no exhaustive analyses of high-temperature culture of actinomycetes. Thus, Saito et al. decided to test on a large scale whether high-temperature culture of actinomycetes can activate silent genes.

First, an in-house library of 3,160 strains of actinomycetes widely isolated from Japanese soil was searched to identify actinomycete strains capable of growing at high temperatures (45 °C). This screening identified 57 actinomycete strains capable of growing at 45 °C. Comparative analysis of metabolites produced by these 57 actinomycetes at room temperature or high temperature revealed that almost half of the strains produced metabolites selectively at 45 °C. As these results were obtained using one type of medium, a set number of incubation days, and one analytical condition using LCMS, it is possible that additional metabolites could be detected by examining a variety of conditions.

Sixteen of the abovementioned strains were randomly selected and cultured on five different media to obtain metabolic profile data according to culture temperature. This analysis resulted in the detection of newly isolated metabolites, metabolites exhibiting enhanced production, metabolites exhibiting diminished production, and metabolites in which production ceased. Plotting the newly identified metabolites according to retention time and molecular weight determined by LCMS, it was expected that most of the metabolites would tend to be relatively polar and of low molecular weight. The results suggested that high-temperature culture induces the production of silent secondary metabolites in certain actinomycetes, and Saito et al. therefore designated these compounds HSMs [72]. Then, the structures of these HSMs are attempted to elucidate (Figure 4b,c).

Gaudimycins A and B (**32**, **33**) were identified as HSMs from *Streptomyces* sp. CA40. Palmu et al. discovered gaudimycins by heterologous expression of the biosynthetic gene clusters *pga* or *cab*, which were silent in the producing strain *Streptomyces* sp. PGA64 or *Streptomyces* sp. H021 relative to another host, *Streptomyces lividans* TK24 [73]. Resistomycin (**34**) [74], resistoflavin (**35**) [75], and tetracenomycin D (**36**) [76] were also identified as HSMs from *Streptomyces* sp. HN66. Carlson et al. reported the production of resistomycin by co-cultivating *Streptomyces* sp. B033 and *Burkholderia vietnamiensis* ATCC BAA-248 [77]. These results suggest that high-temperature culture of actinomycetes activates the production of silent secondary metabolites.

Further investigation of HSMs led to the discovery of three new compounds from different actinomycete strains. The first HSM is murecholamide (**37**), produced by *Streptomyces* sp. AY2 [72]. This substance has a skeleton in which a substructure of desferrioxamine (DFO, **38**), a well-known secondary metabolite [78], is condensed at the end of the steroid skeleton. It is thought that DFO is produced as a result of the activation of a silent gene, whereas the steroid skeleton may be a component of the culture medium. At a concentration of 182 μM, murecholamide exhibits biological activity in inhibiting the migration of HT29 cancer cells. The second HSM is noaoxazole (**39**), which was discovered in *Streptomyces* sp. HR41 [79]. This substance has a chemical structure with a methylated oxazole ring at the end of a chain-like structure. The structure of noaoxazole is similar to that of inthomycin and oxazolomycin [80–81], but the methylated oxazole ring is a relatively valuable structure found elsewhere only in curromycins A and B (**40**, **41**) and neocurromycin [82–83]. As with curromycin A, the end product of noaoxazole is expected to be different, but its discovery has not been reported to date. Noaoxazole has an activating effect on Notch signaling, which may result in inhibition of the malignant transformation of some cancers [84–85]. The third HSM is dihydromaniwamycin E (**42**), which was discovered in *Streptomyces* sp. JA74 [86]. Maniwamycin E (**43**) [87] was also discovered from this strain as a metabolite for which production is enhanced by high-temperature culture. These substances have an azoxy structure, which is relatively rare in the natural environment [88]. The stereochemistry of dihydromaniwamycin E was determined by total synthesis. The biological activities of these maniwamycins were also evaluated, and they showed antiviral activity against influenza (H1N1) virus and the causative agent of COVID-19, SARS-CoV-2. This was the first report of such activity for a natural product having an azoxy structure.

Saito et al. are now interested in determining how and why these HSMs are produced. First, when actinomycetes sense heat stress, repression mediated by heat shock sensor proteins such as HrcA, HspR, and RheA is released. The expression of various genes, such as those encoding heat shock proteins (HSPs), is then activated [89–91]. Lu et al. reported that the heat shock sensor HspR regulates the production of avermectin in *Streptomyces avermitilis* [92]. Further investigation is needed to determine if HSM production is induced after short periods of thermal stress and whether this production is regulated by such heat shock sensors. Although long-term high-temperature culture experiments are employed in this study, reactive oxygen species (ROS) may exert a stronger effect than high temperature. In actinomycetes, several sensor proteins for ROS have been reported (e.g., SoxR and OxyR), which upon sensing ROS, release their repression and activate the expression of various genes [93–95]. Wei et al. reported that the production of validamycin A (**25**) by *Streptomyces hygroscopicus* 5008 could be activated at the transcriptional level by simply adding hydrogen peroxide (H_2_O_2_; which generates ROS) to the culture medium [96]. In addition, there are also reports of cases in which denatured proteins are folded by molecular chaperones in high-temperature environments, and such activity may affect the production of secondary metabolites [97]. Saito et al. are currently attempting to determine how HSMs are produced.

Some studies have reported that secondary metabolites produced by actinomycetes are used physiologically by the producing strains and for communication with other strains. For example, Tenconi et al. reported that under certain conditions, the biosynthesis of undecylprodigiosin (**21**) is always triggered in the dying zone of the mycelial network of *Streptomyces coelicolor* M145 prior to morphological differentiation, right after an initial round of cell death [98]. They hypothesized that under certain circumstances, production of antiproliferative agents could play a role in the genetically programmed death of the producing organism. In addition, Nishiyama et al. suggested that actinorhodin (**8**) produced by *Streptomyces coelicolor* A3(2) functions as an organocatalyst to kill bacteria by catalyzing the production of toxic levels of H_2_O_2_ [99]. They also suggested that living organisms produce and use such metabolites as biocatalysts in nature. Therefore, it is thought that the production of HSMs has some physiological significance. For example, other microbial species reportedly activate energy metabolism and strengthen the cell membrane or walls in high-temperature environments as a means of ensuring thermotolerance [100–102]. As this suggests that HSMs may support thermotolerance, Saito et al. are also currently trying to determine why HSMs are produced.

#### Co-culture

**Figure 5:**
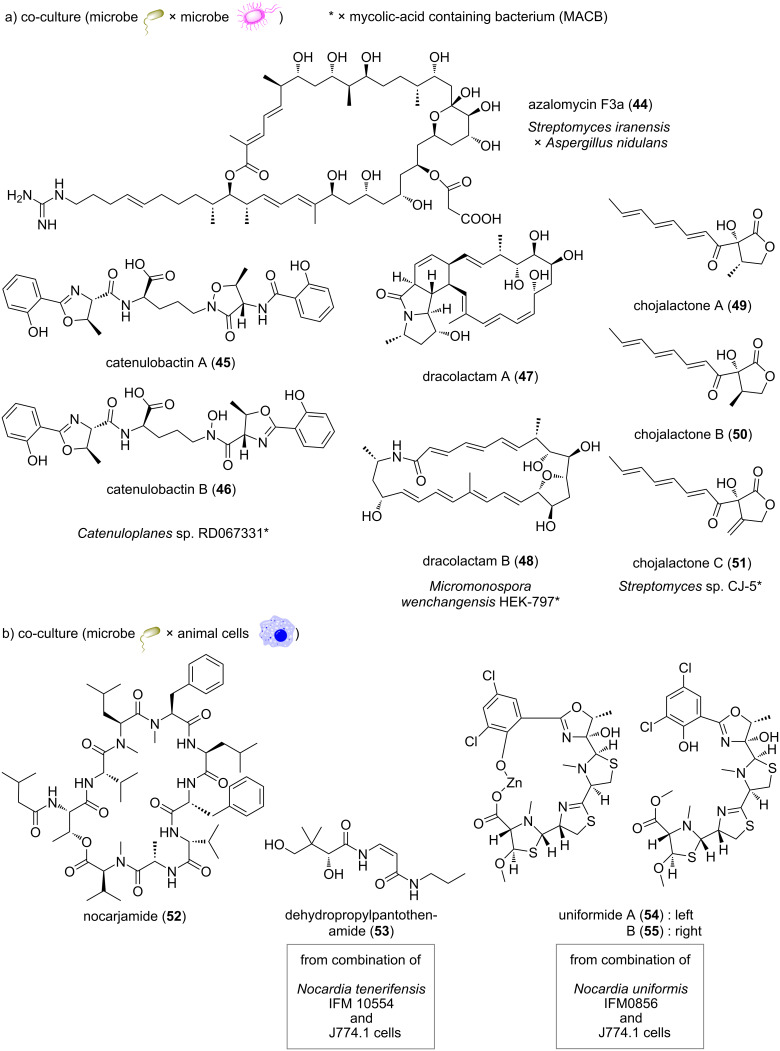
Structures of secondary metabolites obtained by co-culture of actinomycetes with other microorganisms or animal cells.

It is also important to maintain the culture environment in a state close to that of the natural environment in order to activate silent genes in actinomycetes. Actinomycetes mainly inhabit soils, but they are widely symbiotic with plants [103], insects [104], and other organisms [105–106], and there are reports of their secondary metabolites controlling their own physiological functions or functioning as communication molecules [107–108]. Therefore, various studies have attempted to activate secondary metabolite biosynthetic genes under culture conditions that mimic the natural environment. In particular, the co-culture method, in which multiple microorganisms are cultured simultaneously, has been used in many studies examining silent gene activation (Figure 5a).

For example, Krespach et al. reported that *Streptomyces iranensis* secretes azalomycin F3a (**44**) in the presence of *Aspergillus nidulans* [109]. Interestingly, *A. nidulans* also reacted to the presence of azalomycin F3a by producing orsellinic acid, lecanoric acid, and the compounds F-9775A and F-9775B. Lee et al. discovered that secondary metabolism in *Streptomyces coelicolor* A3(2) M145 is activated by iron competition during co-culture with *Myxococcus xanthus* [110]. Co-culture promotes the production of the siderophore myxochelin in *M. xanthus*, which enhances the iron-scavenging capacity of *M. xanthus* and places *S. coelicolor* A3(2) M145 under iron starvation conditions. This chemical interaction activates the actinorhodin (**8**) biosynthetic gene cluster in *S. coelicolor* A3(2) M145. Onaka’s group reported that co-cultivation of *Streptomyces* and mycolic acid-containing bacteria (MACB) activates secondary metabolism [111]. In their method, direct attachment by live MACB cells is thought to alter secondary metabolism in the *Streptomyces* cells [112]. Using this method, Onaka et al. identified a variety of new compounds, such as catenulobactins A and B (**45**, **46**) [113], dracolactams A and B (**47**, **48**) [114], and chojalactones A–C (**49**–**51**) [115]. Finally, although not an example of co-culture, Chevrette et al. found that the potential secondary metabolic capacity differs between soil-derived and insect-derived actinomycetes [116]. Insect-associated *Streptomyces* inhibit antimicrobial-resistant pathogens to a greater degree than soil *Streptomyces*. This suggests that the evolutionary trajectories of *Streptomyces* from the insect microbiome influence their biosynthetic potential and ability to inhibit resistant pathogens. Against this background, a unique case of activation of secondary metabolite biosynthetic genes by co-culture of actinomycetes and animal cells has been reported, which we outline in detail below.

If microorganisms and animal cells are used, one might first imagine the interaction between humans and intestinal bacteria. Indeed, several examples of co-culture of human cells and intestinal bacteria have been reported, and the changes in gene expression on the host side during such co-culture experiments have been well studied [117–118]. On the other hand, unlike intestinal bacteria, pathogenic microorganisms that adversely affect human health also interact with host cells when they invade the body [119–120]. Noting this, Ishibashi and Arai et al., hypothesized that when pathogenic microorganisms invade the body, they are subjected to xenocidal stress from immune cells, which activates silent genes and produces defensive compounds as a countermeasure. Therefore, the pathogenic actinomycetes were co-cultured with J774.1 mouse macrophage-like cells (Figure 5b).

The first compound isolated was nocarjamide (**52**), a cyclic nonapeptide, discovered by co-culturing *Nocardia tenerifensis* IFM 10554^T^ with J774.1 cells [121]. Nocarjamide exhibited biological activity by activating Wnt signaling at an effective concentration of 20–40 μM. Wnt signaling plays an important role in a number of vital processes, such as the formation of various tissues and the differentiation/proliferation of eukaryotic cells [122]. It is interesting that substances that affect Wnt signaling can be produced in response to the interaction between animal cells and microorganisms. In addition, dehydropropylpantothenamide (**53**) was also identified using the same co-culture combination [123]. The second group of compounds identified included uniformides A and B (**54**, **55**), which were discovered by co-culturing *Nocardia uniformis* IFM0856^T^ and J774.1 cells [124–125]. Uniformides were shown to suppress the production of nitric oxide, IL-6, and IL-1β by inhibiting the NF-κB pathway. Because NF-κB signaling plays a central role in the immune response [126], it is interesting that substances that regulate the pathway were produced by co-culture.

Thus, co-culture of animal cells and microorganisms may also be useful as a method for activating silent genes. However, it is important to determine what types of interactions between animal cells and microorganisms activate secondary metabolism in order to understand communication within the human body. For example, co-culture experiments using certain microorganisms have shown that physical contact between live microorganisms is important for the activation of secondary metabolism [112]. In addition, as mentioned above, various substances and changes in the concentrations of metal ions can affect secondary metabolism, and secondary metabolite production may thus be activated by these factors [110].

## Conclusion

Natural products hold great potential as leads in drug development and as useful tools in chemical biology research. Therefore, attempts to obtain novel secondary metabolites by activating the latent metabolic potential of actinomycetes are gaining momentum. In this review, methods used to search for new secondary metabolites, focusing mainly on high-temperature culture of actinomycetes and the co-culture of microorganisms and animal cells, were outlined. The mechanisms by which these silent secondary metabolites are produced should be specific to each method. Elucidation of these mechanisms will directly lead to the development of new silent gene activation methods. In these approaches, there are some disadvantages associated with limited adaptability of microbial species (high-temperature culture) and somewhat complex experimental manipulations (co-culture method). It is important to overcome these disadvantages, broaden the range of adaptable microbial species, and develop a simple method exhibiting high activation efficiency.

The metabolites produced by the activation of silent genes are expected to be physiologically significant. In the case of high-temperature culture or co-culture methods, these compounds may play roles in thermotolerance or communication between different species, respectively. Analyses of the functionality of such metabolites may also lead to the elucidation of new life phenomena. However, in recent years, it has not only become increasingly difficult to obtain novel secondary metabolites from natural products, but applied research using natural products, such as drug discovery studies, has been losing momentum. In drug discovery studies in the field of chemical biology, it may be important to investigate the compounds obtained by silent gene activation. It is expected that compounds useful in a variety of fields will be discovered by making full use of such silent gene activation methods.

## Data Availability

Data sharing is not applicable as no new data was generated or analyzed in this study.
